# A future of AI-driven personalized care for people with multiple sclerosis

**DOI:** 10.3389/fimmu.2024.1446748

**Published:** 2024-08-19

**Authors:** Jelle Praet, Lina Anderhalten, Giancarlo Comi, Dana Horakova, Tjalf Ziemssen, Patrick Vermersch, Carsten Lukas, Koen van Leemput, Marjan Steppe, Cristina Aguilera, Ella Maria Kadas, Alexis Bertrand, Jean van Rampelbergh, Erik de Boer, Vera Zingler, Dirk Smeets, Annemie Ribbens, Friedemann Paul

**Affiliations:** ^1^ icometrix NV, Leuven, Belgium; ^2^ Experimental and Clinical Research Center (ECRC), A Cooperation Between the Max Delbrück Center for Molecular Medicine in the Helmholtz Association and Charité - Universitätsmedizin Berlin, Berlin, Germany; ^3^ Department of Neurorehabilitative Sciences, Casa di Cura Igea, Italy; ^4^ Department of Neurology, Vita-Salute San Raffaele University-Ospedale San Raffaele, Milan, Italy; ^5^ Department of Neurology and Center of Clinical Neuroscience, First Faculty of Medicine, Charles University and General University Hospital, Prague, Czechia; ^6^ Center of Clinical Neuroscience, Department of Neurology, University Clinic Carl Gustav Carus, TU Dresden, Dresden, Germany; ^7^ Univ. Lille, InsermU1172 LilNCog, CHU Lille, FHU Precise, Lille, France; ^8^ Institute of Neuroradiology, St. Josef Hospital, Ruhr-University Bochum, Bochum, Germany; ^9^ Athinoula A. Martinos Center, Department of Radiology, Massachusetts General Hospital, Charlestown, MA, United States; ^10^ Department of Neuroscience and Biomedical Engineering, Aalto University, Espoo, Finland; ^11^ Department of Computer Science, Aalto University, Espoo, Finland; ^12^ European Charcot Foundation, Brussels, Belgium; ^13^ SYNAPSE Research Management Partners, Madrid, Spain; ^14^ Nocturne GmbH, Berlin, Germany; ^15^ AB Science, Clinical Development, Paris, France; ^16^ Imcyse SA, Liège, Belgium; ^17^ Bristol-Myers Squibb Company Corp, Princeton, NJ, United States; ^18^ F. Hoffmann-La Roche Ltd., Product Development Medical Affairs, Neuroscience, Basel, Switzerland; ^19^ Experimental and Clinical Research Center (ECRC), Charité - Universitätsmedizin Berlin, Corporate Member of Freie Universität Berlin and Humboldt-Universität zu Berlin, Berlin, Germany; ^20^ Max Delbrück Center for Molecular Medicine in the Helmholtz Association (MDC), Berlin, Germany; ^21^ Neuroscience Clinical Research Center (NCRC), Charité - Universitätsmedizin Berlin, Corporate Member of Freie Universität Berlin and Humboldt-Universität zu Berlin, Berlin, Germany; ^22^ Department of Neurology with Experimental Neurology, Charité - Universitätsmedizin Berlin, Corporate Member of Freie Universität Berlin and Humboldt-Universität zu Berlin, Berlin, Germany

**Keywords:** multiple sclerosis, personalized medicine, disease progression, prognosis, diagnosis, AI, data

## Abstract

Multiple sclerosis (MS) is a devastating immune-mediated disorder of the central nervous system resulting in progressive disability accumulation. As there is no cure available yet for MS, the primary therapeutic objective is to reduce relapses and to slow down disability progression as early as possible during the disease to maintain and/or improve health-related quality of life. However, optimizing treatment for people with MS (pwMS) is complex and challenging due to the many factors involved and in particular, the high degree of clinical and sub-clinical heterogeneity in disease progression among pwMS. In this paper, we discuss these many different challenges complicating treatment optimization for pwMS as well as how a shift towards a more pro-active, data-driven and personalized medicine approach could potentially improve patient outcomes for pwMS. We describe how the ‘Clinical Impact through AI-assisted MS Care’ (CLAIMS) project serves as a recent example of how to realize such a shift towards personalized treatment optimization for pwMS through the development of a platform that offers a holistic view of all relevant patient data and biomarkers, and then using this data to enable AI-supported prognostic modelling.

## 1 The heterogeneous disease course of multiple sclerosis

Multiple sclerosis (MS) is a devastating immune-mediated disorder of the central nervous system (CNS) resulting in progressive disability accumulation in most individuals affected (1, 2). MS imposes a significant burden on patients, affecting all aspects of their life, and additionally, it poses a significant challenge to society as with growing disability, indirect expenses (productivity losses associated with sick absence, inability to work, and early retirement) and care costs rise substantially (3).

The classical view on MS describes different clinical subtypes, with relapsing-remitting MS (RRMS) being the most common form, occurring in 85% of patients (National MS Society). Patients with RRMS experience neurological exacerbation (relapses) as well as intermittent periods of remission in which they remain clinically stable. Relapses can either recover completely or leave persistent clinical disability, referred to as Relapse Associated Worsening (RAW). Among these patients, approximately two-thirds progress to secondary-progressive MS (SPMS) (4). In contrast to RRMS, the disease course of patients with SPMS or primary-progressive MS (PPMS, 15% of MS patients) is mainly driven by a gradual worsening of disability in the absence of relapse activity (5).

Recent research has challenged this classical view of distinct MS subtypes, as they may not sufficiently account for the large spectrum of multifaceted clinical phenotypes and disease courses as well as sub-clinical disease variability (6). This disease heterogeneity is further complicated by a high prevalence of comorbidities and multi-pharmacy in MS. Data from the NARCOMS registry suggested that, at the time of MS diagnosis, 35% of MS patients suffer physical comorbidities while 18% reported a psychiatric comorbidity (7, 8). Additionally, accumulation of clinical disability independent of acute inflammatory relapses - commonly referred to as Progression Independent of Relapse Activity (PIRA) (9) - was found to occur in any of the classical MS subtypes, including RRMS, and at any stage of the disease (10, 11). Most importantly, in a substantial proportion of people with MS (pwMS), PIRA occurs already very early on, and this is associated with worse long-term outcomes (2). Recent studies have also shown that PIRA gradually becomes the dominant driver of disability worsening as the disease progresses (9).

While new insights into PIRA continue to be unraveled, exact criteria of how to define, assess, and monitor PIRA are still lacking. Several definitions have been put forward, but these focus mainly only on measuring disability worsening by means of the Expanded Disability Status Scale (EDSS) and Confirmed Disability Worsening (CDW) (2). Relying solely on EDSS or CDW to describe PIRA, however, seems to be insufficient as (i) there are heterogeneous symptoms and disease aspects contributing to disability worsening and MS severity, and (ii) this omits sub-clinical processes such as compartmentalized inflammation, chronically active (smouldering) lesions, diffuse normal-appearing matter damage (12, 13), as well as brain (14) and spinal cord atrophy (15, 16). Such processes seem to represent relevant substrates of (silent/smouldering) disease progression even during early stages and to contribute to enhanced long-term disability worsening in pwMS (17). In this regard, the topographical disease model proposed by Krieger et al. may facilitate the interpretation of the clinical course revision, providing a unified visualization across phenotypes, while providing insights in the interplay between the distinct processes of relapse activity and progression, and accounting for latent variables such as relapse localization, frequency, severity, recovery and progression rate (18). Additionally, this model was recently validated in terms of brain MRI markers (19). Aligning with this model, individuals deemed neurologically normal in early MS (e.g., with an EDSS score of 0) demonstrated subtle deficits in high-challenging motor tasks (20) and often have fatigue (21) and cognitive impairments (22). The former was also shown to correlate with imaging markers of disease burden and brain reserve, challenging traditional severity definitions and underscoring the importance of looking beyond standard clinical measures such as the EDSS (20).

## 2 A changing landscape in treatment strategies

The heterogeneity in disease progression among individuals with MS (both clinically and sub-clinically) contributes to a high diversity in treatment responses across pwMS (23). As there is no cure available yet for MS, the primary therapeutic objective is to slow down disability progression and to reduce relapses as early as possible during the disease to maintain and/or improve the health-related quality of life (24).

To this end, all regulatory-approved disease-modifying treatments (DMT) have shown their worth in preventing relapses during the few years of the clinical trial in which their efficacy was evaluated. However, the impact on the long-term accumulation of disability and chronic subtle disease processes was often limited as even the most effective DMTs available were only able to mitigate the short-term risk of disability progression by 30-42% (25). A recent review from Gasperini et al. emphasizes how dire the situation really is, indicating that only 30- 40% of patients receiving a DMT remain stable over a period of 5 to 7 years, and only up to 10% over a period of 7 to 10 years after initiating DMT (26).

Despite the approval of ±20 different DMTs by the European Medicines Agency (EMA) and the US Food and Drug Administration (FDA) (27, 28), concerns about side effects and efficacy might discourage many pwMS from initiating a high-efficacy DMT therapy (29, 30), an issue further aggravated by therapeutic inertia (31). Additionally, those who do receive a DMT usually start with one of the less effective but well-established therapies due to their minimal side effects (32). Traditionally, it’s only when these well-established DMTs fail to prevent relapses and disability progression, that the treatment is escalated to a higher-efficacy treatment, which usually is more expensive, might have more pronounced side effects, and is potentially more challenging to administer (oral and injectables versus infusions) (33). However, multiple studies support the observation that reducing the accrual of neurological damage in the initial stages of the disease potentially improves overall clinical outcomes throughout the patient’s lifespan when employing early intervention with higher efficacy DMT (34–38). Additionally, DMTs were shown to be more efficacious, and side effects less likely to occur in younger patients (39). Taken together, these studies question the traditional treatment escalation paradigm which is therefore nowadays considered outdated by most physicians. Instead, current thinking emphasizes the potential advantages of early initiation of high-efficacy DMTs, indicating the need for and the significance of an early MS diagnosis, proactive monitoring to detect disease activity early, and shared decision-making as crucial elements in patient care (32, 40).

Additionally, given the shortcomings of current DMTs to halt long-term disability accumulation, a next generation of DMTs might focus more on the silent progression of the disease. A first novel category of DMTs in this regard are potentially the Bruton tyrosine kinase inhibitors. This new class of drugs might become the first to target both acute inflammatory relapses as well chronic inflammatory processes in the CNS thought to drive disability accumulation (41). In this context, especially the early recognition of individuals prone to developing PIRA will be essential. A better understanding of PIRA and RAW as well as their interplay, combined with data-driven prognosis, will enhance the selection of current and future DMTs and allow to treat patients beyond just relapse activity. Nevertheless, certain variables pose challenges to the trajectory of precision medicine and treatment optimization on an individual level. While there are guidelines on the use of DMTs in MS (24), these are all based on expert judgment and differ across countries, even within the EU (28, 37). This variance extends to therapy selection post-diagnosis or during follow-ups, driven by perceived levels of clinical and subclinical disease activity and progression.

## 3 Precision medicine enables treatment optimization

Accumulating evidence suggests that the reactive treatment of lesion activity is insufficient, negatively impacting long-term patient outcomes (42). In the complex landscape of MS treatment, an increasing acknowledgment of disease heterogeneity and underlying disease mechanisms underscores the imperative for a paradigm shift toward proactive, data-driven precision medicine (43). However, despite its promise, such data-driven approaches come hand in hand with substantial challenges.

The understanding of the complex and heterogeneous underlying neuropathology of MS is still limited. The adoption of precision medicine in MS is further complicated by the chronic nature of the disease, exhibiting variable courses over time. Consequently, given the longitudinal disease aspect, one must account for the fact that data might be incomplete at times, particularly in routine practice. In addition, the influence of comorbidities adds another layer of complexity (44). Various biomarkers are deemed relevant for their role in identifying diverse MS aspects and patterns of progression in MS, aiding diagnosis, prognosis, and treatment selection (45). However, they might not capture the full complexity of MS and their interpretation requires a nuanced understanding of the disease context. Moreover, the heterogeneous nature of MS challenges the development of universally applicable biomarkers and complicates the tracking of different treatment effects on an individual basis (46).

Notably, with a variety of treatment options being available (27, 28), emerging biomarkers, including liquid and imaging markers, have shown potential in monitoring treatment efficacy (45, 47). However, the validation, availability, and implementation of biomarker assessments in real-world clinical practice is often still missing as this differs significantly from their application in clinical trials. Moreover, biomarkers that demonstrate both sensitivity and specificity in the context of progressive MS are still lacking (47). While early diagnosis and prognosis modelling are pivotal for timely and effective treatment initiation, the ability to clearly define and disentangle disability accumulation attributed to RAW or PIRA will be key to optimizing individual treatment over the course of the disease.

**Figure 1:**
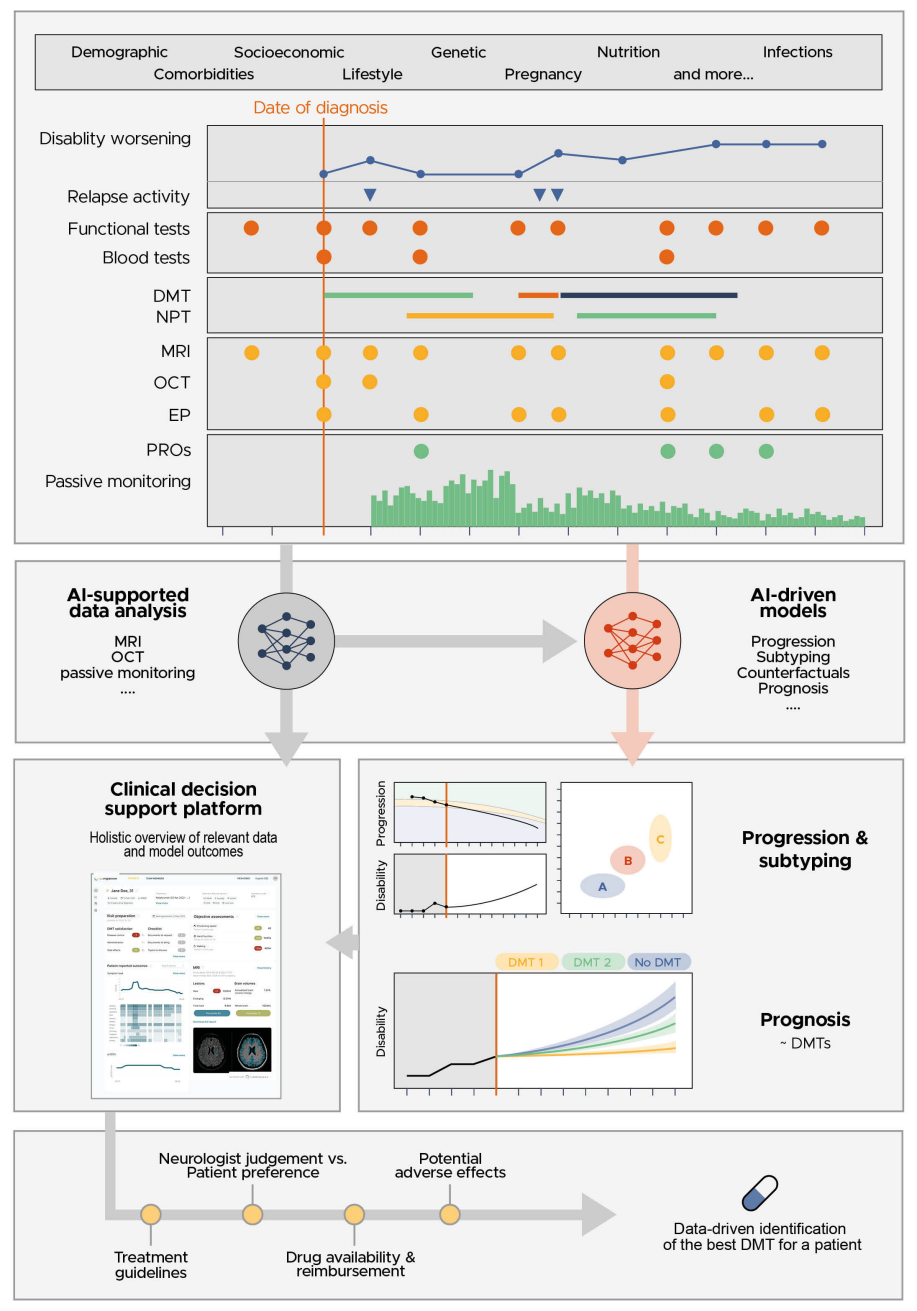
A clinical decision support tool should be capable of visualizing the very heterogenous MS patient data, the AI-supported analysis of this data and the outcome of prognostic models using this data, enabling a data-driven discussion between the neurologist and patient to identify the best DMT for the patient.

Advancements in artificial intelligence (AI) can offer enhanced and data-driven support by considering longitudinal data on multiple biomarkers simultaneously and subtyping patients more accurately. In particular, this can include biomarkers more related to PIRA such as motor dysfunction beyond EDSS (2, 48), optical coherence tomography (49–51), magnetic resonance imaging markers predictive of disability worsening such as brain atrophy (14), slowly expanding lesions and paramagnetic rim lesions (52–54) and cognitive impairment (55–57), as well as subjective markers [i.e. patient-reported outcomes (PROs) such as quality of live (58, 59)]. We believe that a holistic overview of the patient will be crucial to avoid overlooking relevant information, including both existing and new biomarkers as our disease understanding evolves further (**Figure 1**).

Such transformative approaches hold the potential to significantly enhance treatment strategies and extend the adjusted quality of life years for individuals with MS. Nevertheless, the current landscape is still fragmented, often focusing on singular aspects or biomarkers rather than adopting a more holistic and comprehensive approach. Data strategies to reduce the level of heterogeneity, particularly improving data harmonization by means of a common data model, are wishful to guarantee standardization in clinical decision making (60). However, the implementation of such initiatives is still in the early stages. Care pathways for pwMS are also not commonly standardized and while some diagnostic and treatment guidelines and recommendations are available (1, 61, 62), the assessment of relevant outcomes may not always be sufficiently covered and integrated into the routine clinical workflow (63). A modular-integrative framework of digital patient pathways for MS management and treatment is needed, which should incorporate AI, data harmonization and review relevant research concerning the use of pathways in healthcare (64, 65). Although initial evidence of acting upon AI-driven MRI biomarkers has indicated to improve patient outcome (66), the evaluation of impact in real-world practice and evidence on whether acting upon data-driven models and biomarkers truly improves the quality of life for patients with MS are crucial components that demand more attention in the pursuit of effective precision medicine strategies for MS.

## 4 Clinical impact through AI-assisted MS care

A data-driven and personalized clinical decision support tool is urgently needed for MS, to prevent and slow down disease progression more efficiently via optimizing treatment. The EU-funded ‘Clinical Impact through AI-assisted MS Care’ (CLAIMS, www.claims.ms) project aims to address this need. The project will develop, validate and seek regulatory approval for an AI-driven clinical decision-support platform, which offers the MS care team a holistic view of the patient through the visualization of all relevant patient data and the prognosis on the expected disease trajectories under different treatment regimens.

Initially, the project focusses on the development and optimization of these prognostic models via the use of retrospectively collected clinical routine data in combination with clinical trial data. A detailed description of this retrospective multi-center observational study (called RECLAIM) is accessible via ClincialTrials.gov. This study aims to collect and harmonize both clinical and subclinical data and store it in a central database on a secure cloud environment. Data harmonization will be following the common data model proposed in Parciak et al. (67), but kept to the minimum necessary as we aim to stay as close as possible to the real-world clinical setting and to ensure the clinical relevance.

The combination of real-world with clinical trial data is an important aspect of the study. Clinical trial data is very homogeneous and highly curated, making it an ideal dataset to develop AI-driven prognostic models. For instance, MRI scans obtained in clinical trials adhere to a standardized protocol, include all necessary sequences, and ensure follow-up scans within a specific timeframe. In contrast, MRI scans acquired in a real-world setting frequently don’t meet these requirements (68, 69). As the CLAIMS project aims to create AI-based prediction models applicable in real-world clinical settings, it is crucial to also incorporate routine care data in the development and validation phases. By combining both types of data, we aim to achieve an extensive dataset that leverages the strengths of both types of data ensuring applicability in a routine clinical care setting where confounding factors (e.g., comorbidities), low quality data and missing data are common (70, 71).

The focus will be on modelling disease progression. Disease progression models often have strong assumptions about the monotonicity of disease progression processes, the missingness model and associated completeness of the data, the longitudinal regularity of the observations, and homoscedastic noise characteristics of the measurements. Due to the different MS subtypes, and relapse and recurrence events, many of these assumptions do not hold in a MS setting. Furthermore, when using clinical observational data, data points are missing-not-at-random, both because patients often miss their appointments, but also because certain examinations (clinical assessments, MRI, etc) are performed as a function of patient presentation. Tackling this requires us to explore applicability of advanced and appropriate models of data imputation, and from generative models that explicitly model the causal relationships of the observations.

Contrary to clinical research trials where patients are assigned to a treatment or placebo arm at random, in an observational setting, DMTs are given to patients according to guideline recommendations and patient presentation. Observational data is thus biased by these guidelines, and appropriate measures are needed to control for this bias. Causal inference mechanisms via counterfactuals allows one to model such observational data and predict what the potential outcome would have been under a counterfactual treatment. By disentangling causes and effects, one gains a clearer understanding of the underlying biological or pathological markers that are predictive of the observed effect and outcome. This enables a more grounded clustering of patients (e.g., what are the patient characteristics that predict drug efficacy), providing an explanation of the optimal therapeutic inference (e.g., what is the biological reason why a certain drug is optimal for a specific patient). While some of these challenges have been addressed in highly controlled randomized clinical research environments, solving them using an observational experimental setup would allow one to exploit large amounts of data while ensuring the models remain accurate when deployed in a real-world environment where the aforementioned problems exist. Observational studies using real-world data allow for more heterogeneous and comprehensive cohorts, thereby elevating external validity and supplying valuable insights to guide treatment approaches (69).

**Figure 2:**
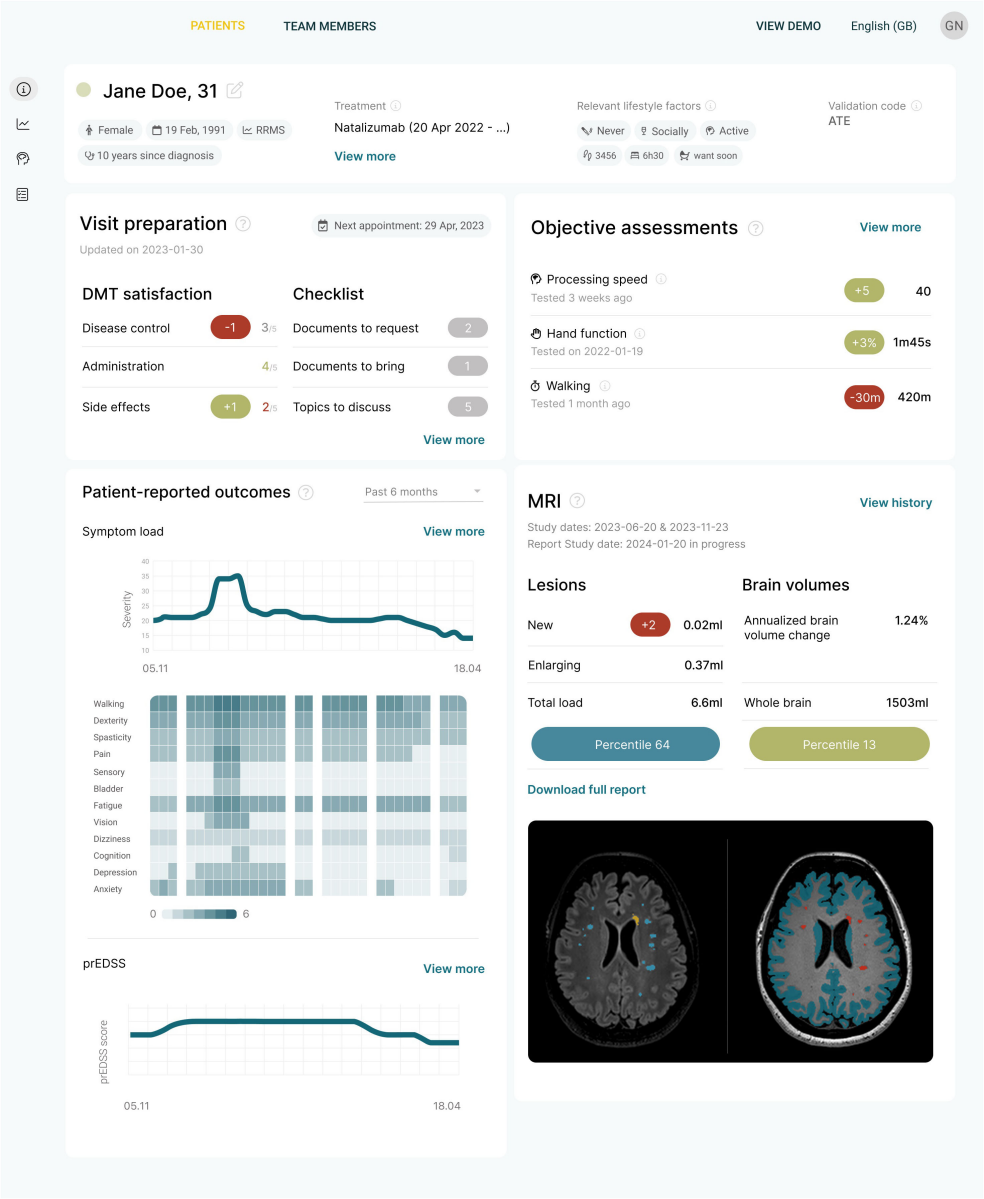
The first iteration of the clinical decision support platform being developed in the CLAIMS project. It offers a concise overview of the most important data for making a clinical decision.

At the time of writing this paper, the first version of the CLAIMS platform was already available, building upon a regulatory cleared AI solution for brain MRI quantification, a patient app for pwMS and a regulatory cleared AI solution for optical coherence tomography (OCT) quantification (72–75), but without the prognostic models (**Figure 2**). The complete clinical decision support platform, including the prognostic models, will be included in prospective clinical trial (called PROCLAIM), designed to obtain regulatory approval, and bringing it to the market as soon as possible. Meanwhile, the platform will be iteratively improved as new biomarker data becomes available and models are further refined. This iterative approach ensures that the CLAIMS project achieves true clinical impact for patients sooner rather than later.

## 5 Digital health and how this support prognosis

The CLAIMS project is exploring an additional avenue for the identification of promising markers of disease progression by capturing digital biomarkers using digital health tools. A first set of digital health tools includes AI solutions tailored for the quantification of brain MRI scans (74, 75). Notable advancement of these tools’ accuracy, in combination with rigorous technological, workflow, clinical and even initial health economic validation makes that this solution steadily gains recognition as standard of care. In the United States, this trend towards embracing AI-based brain MRI quantification is further exemplified by the recent provision of two new Current Procedural Terminology (CPT) codes. Evidence has shown that by using such a solution, disease activity can be detected up to 3 years earlier with a potentially significant impact on treatment decisions (66).

Patient apps, another major trend in the digital health tools, could enhance the early detection of disease progression in pwMS and allow monitoring disease progression in between visits with their treating physician. This can be achieved by monitoring symptoms and disability progression through capturing patient-reported outcomes (PROs), through passive monitoring of various markers (activity, sleep, vital signs, …) or through the digital administration of tests assessing for example cognition, vision, mobility, etc. (76, 77). In addition, these tools can play an important role in increasing and monitoring medication adherence, improving a patient’s lifestyle through creating awareness, and to educate and empower patients in managing their disease better. As such, disease monitoring via digital health tools provides a dynamic, more continuous, and more nuanced understanding of disease progression.

Development of such tools poses a socio-technical challenge. Any tool which aims to obtain regulatory clearance for use in a clinical setting will need to obtain sufficient technical and clinical evidence, which is often a long and laborious process. A bigger challenge, however, is patient adoption and thereafter adherence in using the tools. Concerns on data security and privacy need to be adequately addressed and simultaneously, it needs to be very clear to patients that they will benefit from enhanced care and personalized interventions driven by a more holistic understanding and monitoring of their health status and disease progression. CLAIMS aims to address this by empowering and educating patients on the need to better monitor their disease. In this light, the patient app used in CLAIMS is positioned as a companion app, available to support the patient as needed, focusing on topics of interest to the patient, rather than mandating the app usage. Actively involving patients and capturing their feedback on the app utilization, whether via real-world usage or within a clinical study setting, will contribute valuable insights, allowing to further refine the tools and ultimately, the clinical decision support platform.

Besides patient adherence, integration into routine clinical workflows poses another challenge. To address this, the clinical decision support platform in CLAIMS aims to keep the steps of platform adoption to a bare minimum. It aggregates all of a patient’s data, including data from the patient app, from the AI-driven MRI analysis and from the AI-driven OCT-analysis. While the full datasets and analyses will be available via this platform, the main dashboard focusses on providing a holistic overview of all clinically actionable measures and markers. While this is rather straightforward for subjective and episodic data such as with questionnaires or simple tests captured via the patient app, this will be harder to achieve for data from passive monitoring. The latter is known to generate large longitudinal datasets where AI algorithms are needed to identify subtle patterns and disease subtypes, and to predict trajectories.

Patient-reported outcomes (PROs) represent a unique occasion to involve patients using digital health tools and measure the impact of health care on outcomes that hold utmost significance to pwMS. However, the variety of PRO measures available and the absence of standards across different healthcare centers and countries present a considerable challenge (58). The recently established initiative ‘Patient-Reported Outcomes for Multiple Sclerosis’ (PROMS), consisting of an interdisciplinary, international network of different stakeholders, addresses the challenge of creating PRO measures that meet the diverse needs of all parties involved to enhance the influence of both scientific research and patient perspectives on the lives of pwMS (59). In this context, digital health tools enable meaningful assessments, but patient satisfaction can influence assessment compliance and indirectly affect outcome measures. To assess patient satisfaction with digital tools, patient-reported and expert-reported experience measures (PREM) should be collected in parallel (78).

## 6 The road ahead

As our understanding of MS increases, it becomes evident that we should go beyond making treatment decisions solely based on relapses, EDSS progression and lesion activity and move towards proactively treating pwMS for the best possible prognostic outcome. A focus on maintaining/improving health-related quality of life and slowing down disease progression and disability worsening - also independent of relapse activity - has sprouted a clear need for data-driven and personalized clinical decision support tools in MS. Such tools are crucial to administer the right drug to the right patient at the right time to preserve long-term neurological function while minimizing side effects. However, such solutions require well validated biomarkers and models that clearly link to the specificity of the disease course and outcome at individual patient level and can be easily implemented along the clinical care path of the patient.

The CLAIMS project aims to develop such a data-driven and personalized clinical decision support tool while addressing the posed challenges. Biomarker validation and model building will be performed in the retrospective RECLAIM study using both real world data and data from clinical trials. Subsequently, the prospective PROCLAIM study will evaluate the envisioned platform in daily clinical routine, evaluating feasibility and impact on patient care pathways and patient outcome. As such the project will generate a platform for daily clinical routine that provides a holistic view of each patient including existing and novel biomarker assessments to better monitor relapse related disability worsening and progression independent of relapse activity. Driven by deep-learning-based disease subtyping and progression models, the platform will allow the estimation of individual disease trajectories and as such contribute to the urgent need of a more pro-active and data-driven precision medicine in MS care.

## Data Availability

The original contributions presented in the study are included in the article/supplementary material. Further inquiries can be directed to the corresponding author.
